# Comparative Study on the Phenolic Fingerprint and Antioxidant Activity of Strawberry Tree (*Arbutus unedo* L.) Leaves and Fruits

**DOI:** 10.3390/plants11010025

**Published:** 2021-12-22

**Authors:** Irena Brčić Karačonji, Karlo Jurica, Uroš Gašić, Aleksandra Dramićanin, Živoslav Tešić, Dušanka Milojković Opsenica

**Affiliations:** 1 Analytical Toxicology and Mineral Metabolism Unit, Institute for Medical Research and Occupational Health, Ksaverska Cesta 2, 10000 Zagreb, Croatia; 2 Faculty of Health Studies, University of Rijeka, Viktora Cara Emina 5, 51000 Rijeka, Croatia; 3 Special Security Operations Directorate, Ministry of the Interior, Ulica grada Vukovara 33, 10000 Zagreb, Croatia; juricakarlo@gmail.com; 4 Institute for Biological Research “Siniša Stanković”—National Institute of Republic of Serbia, University of Belgrade, Bulevar despota Stefana 142, 11060 Belgrade, Serbia; 5 Faculty of Chemistry, University of Belgrade, Studentski trg 12-16, 11158 Belgrade, Serbia; akosovic@chem.bg.ac.rs (A.D.); ztesic@chem.bg.ac.rs (Ž.T.); dusankam@chem.bg.ac.rs (D.M.O.)

**Keywords:** *Arbutus unedo*, Ericaceae, fruit, leaf, phenolic profile, antioxidant, UHPLC-LTQ Orbitrap MS

## Abstract

The strawberry tree (*Arbutus unedo* L., Ericaceae family) is an evergreen Mediterranean shrub whose leaves and fruits are used in traditional medicine due to their antioxidant, antimicrobial, antidiabetic, diuretic, and antiproliferative properties. The health benefits are mainly attributed to the presence of phenolic compounds. The aim of this study was to compare the phenolic profiles, total phenolic content (TPC), and radical scavenging activity (RSA) of *A. unedo* leaves and fruits collected at two locations in Croatia. Phenolic profiles were identified using an ultra-high-performance liquid chromatograph (UHPLC) coupled with a hybrid mass spectrometer (LTQ Orbitrap MS). TPC was determined by Folin–Ciocalteu’s assay, while RSA was investigated using DPPH reagent. A total of 64 phenolics (60 and 42 compounds in leaves and fruits, respectively) were identified. Hyperoside and flavan-3-ols were predominant compounds in leaves, while gallocatechin and catechin were the major compounds found in fruits. To the authors’ knowledge, 16 and 5 phenolics in leaves and fruits, respectively, were reported for the first time. Principal component analysis (PCA) showed that UHPLC-LTQ Orbitrap MS could be used to identify which phenolics were able to discriminate samples regarding plant tissue and geographical origin. TPC in leaves and fruits were in the ranges of 67.07–104.74 and 16.78–25.86 mg gallic acid equivalents (GAE)/g dried weight (dw), respectively. RSA for leaves and fruits were in the ranges of 408.92–430.98 and 74.30–104.04 μmol Trolox equivalents (TE)/g dw, respectively. The number of identified phenolics was lower in fruits compared to leaves. Such a large number of bioactive phenolics identified and the strong antioxidant activity pointed to *A. unedo* as a promising health-promoting plant and natural food preservative.

## 1. Introduction

Secondary metabolites are bioactive compounds generally involved in plant defence against ultraviolet radiation, diminished water supply, or aggression by pathogens [1]. These substances, in addition to nutritional value, also have a protective effect in reducing the risk of various diseases: they are helpful in the cellular defence and activation of enzyme systems for detoxification, have antioxidant activity, reduce inflammation, and exhibit antimutagenic and antitumor activity [2]. Due to their strong antioxidant properties, there is increased interest in the identification of phytochemicals in Mediterranean wild plants such as evergreen shrub strawberry tree (*Arbutus unedo* L., Ericaceae family) [3].

Phytochemicals, mainly phenolic compounds, that influence human health due to their antioxidative, antimicrobial, anti-inflammatory, and antiradical properties are present in all parts of *A. unedo* [4]. Strawberry tree leaf infusion has diuretic, astringent, and uroantiseptic properties, and is used in folk medicine for treating hypertension, diabetes, and inflammation [4,5]. Strawberry tree fruits, which fully ripen in autumn, are most commonly used to make marmalade, jam, or liquor [4]. The fruits exhibit antiseptic, laxative, and diuretic properties, and are used in folk medicine for the treatment of cardiovascular, urological, dermatological, and gastrointestinal problems [4,6,7]. The increased interest in replacing synthetic antioxidants from food with natural ones has supported research on plants with a high content of bioactive compounds as a source of new antioxidants.

Considering all of the above, there is a need to expand the knowledge on the phytochemicals in *A. unedo* in order to discover new natural antioxidant sources. According to the literature, until now there have been only studies that determined the limited numbers of phenolic compounds in leaves (12–40 phenolics) [8,9,10,11,12,13] and fruits (8–42 phenolics) [7,8,9,10,11,13,14,15,16,17,18,19,20,21,22] using different analytical techniques such as gas chromatography-mass spectrometry (GC-MS) [14]; high-performance liquid chromatography (HPLC) coupled with either mass spectrometry (MS) [7,10,11,12,15,16,19,21], high-resolution MS (HRMS) [13], or diode array detection (DAD) [9,16,19,20,22]; and ultra-performance liquid chromatography (UPLC) coupled with tandem mass spectrometry (MS/MS) [18]. Among all phenolic compounds, flavonoids were the dominant compounds. Apart from a general lack of literature data concerning complete phenolic profiles, only a few studies were carried out with regard to the phenolic profiling of *A. unedo* leaves using HPLC-MS [8], HPLC-MS/MS [10,11,12], or LC-HRMS [13,23], techniques that enable the unambiguous identification of phenolic compounds.

To obtain a more comprehensive characterization of *A. unedo*, this study provided data on phenolic fingerprint, total phenolic content, and antioxidant and antiradical activity of leaves and fruits. Efficiency of phenolic extraction from leaves and fruits using water and methanol was also evaluated. As the content of phenolic compounds is greatly affected by environmental factors (soil type and climatic factors) [23,24,25], leaf and fruit samples were collected from different geographical locations in order to establish a more representative phenolic profile. The aims of this study were to determine and compare the detailed phenolic profile in strawberry tree leaf and fruit extracts, as well as total phenolic content and radical scavenging activity, in order to evaluate this plant as a source of antioxidants. To the best of our knowledge, this is the most comprehensive study on phenolics in *A. unedo* up to now, and the first report of an identification of up to 16 compounds in *A. unedo* leaf or fruit samples. In addition, the relation between activity and structure of quantified phenolics in *A. unedo* was established for the first time.

## 2. Results and Discussion

Although extensive studies of the total content and antiradical activity of bioactive compounds in *A. unedo* have been carried out, the phenolic qualitative and quantitative data are still insufficient and incomplete. The phenolic profile of *A. unedo* fruits was studied somewhat more comprehensively than the leaf profile using LC-MS. Several authors [16,17,20,22,23] determined 8–17 phenolics in fruits. Pallauf et al. [7] and Maldini et al. [13] determined 22 and 17 phenolics, respectively, mostly as derivatives; while Tavares et al. [11], Mendes et al. [10], and Salem et al. [21] determined 30, 25, and 17 phenolics in fruits, respectively, dominantly in the form of gallic acid derivatives. In studies by Mosele et al. [18] and El Cadi et al. [19], 40 and 42 phenolics in total were identified, respectively, which was in accordance with our study, in which 42 phenolics were identified in fruits (Table 1), 5 of which were identified for the first time.

Previous studies concerning the composition of phenolics in *A. unedo* leaves usually included a limited number of compounds [8,9,11,26], and there is a general lack of literature data concerning complete phenolic profiles. Only a few studies were carried out with regard to the phenolic profiling of *A. unedo* leaves using HPLC-MS [8], HPLC-MS/MS [10,11,12], or LC-HRMS [13,23], techniques that enable the unambiguous identification of phenolic compounds. These authors determined 12–40 phenolics in leaves, which was far less than the 60 phenolics identified in leaves in our study.

### 2.1. Qualitative Phenolic Profile of A. unedo Leaves and Fruits

**Figure 1:**
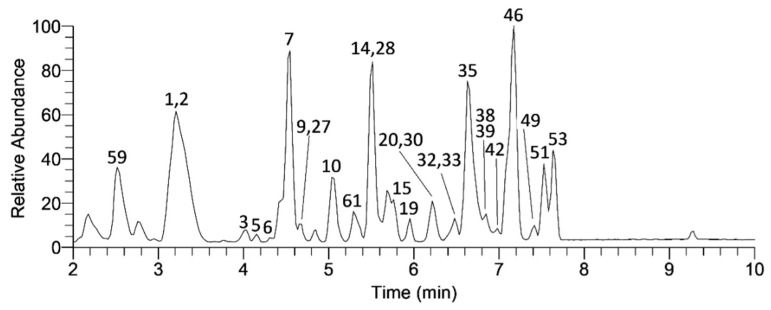
Base peak chromatogram of the leaf sample from Mali Lošinj Island. The peak numbers correspond to Table 1.

The present study gave insight into the profile of the *A. unedo* leaves and fruits using an UHPLC-LTQ OrbiTrap MS, which resulted in the identification of a significant number (*n* = 64) of phenolic compounds (60 and 42 compounds (Table 1) in leaves and fruits, respectively). As expected, the leaf samples of *A. unedo* were richer in phenolic compound content than the fruit samples. As an example, the base peak chromatogram for the phenolic fraction of the leaf sample from Mali Lošinj is shown in Figure 1.

Some flavonoid aglycones (morin, naringenin, myricetin, and kaempferol) were found only in leaf samples, while other flavonoid glycosides (quercetin 3-*O*-galactoside, kaempferol 3-*O*-glucoside, and quercetin 3-*O*-rhamnoside) were found only in fruit samples. Regarding phenolic acids, gallic acid was identified only in fruits, while protocatechuic acid and chlorogenic acid were detected only in leaves. Morin was detected only in methanolic leaf extract from Mali Lošinj, while keampferol 3-*O*-glucoside (astragalin) was detected only in water fruit extract from the same location.

All of the identified compounds represented three structurally distinct groups: phenolic acids and their derivatives (26 compounds), flavonoids and their derivatives (32 compounds), and the other phenolic compounds, including arbutin derivatives (6 compounds). To the authors’ knowledge, 16 and 5 compounds in leaves and fruits, respectively, were reported for the first time (Table 1). Twenty-eight phenolics were identified using available analytical standards (25 and 20 compounds in leaves and fruits, respectively. In the absence of phenolic standards, identification was based on the search for the [M–H]^−^ deprotonated molecule combined with the interpretation of its MS^2^, MS^3^, and MS^4^ fragmentations. Using this approach, we identified a large number of phenolic acid glycosides and flavonol glycosides.

#### 2.1.1. Phenolic Acids and Their Derivatives

In the investigated extracts, examination of mass spectra revealed various phenolic acid glycosides with characteristic fragmentation by losing the sugar unit (162 Da–hexosyl or 132 Da–pentosyl). For example, compound **6** at 4.31 min and 331 *m*/*z* was identified as gallic acid hexoside. The MS^2^ base peak of this compound was at 169 *m*/*z* (loss of 162 Da), and the MS^3^ base peak at 125 *m*/*z*, which was obtained by further loss of the CO_2_ group (44 Da). In addition to this compound, several other gallic acid esters with some organic (quinic and shikimic) acids were found in *A. unedo* extracts. Thus, compound **2** at 3.21 min, with a molecular ion at 343 *m*/*z*, MS^2^ base peak at 191 *m*/*z* (obtained by elimination of galloyl group), and MS^2^ secondary peak at 169 *m*/*z* (corresponding to mass of deprotonated gallic acid) was tentatively identified as galloylquinic acid. According to Jardim et al. [27], such a compound was specific to the *A. unedo* plant, but using only mass spectrometry, it could not be determined whether it was a 3-*O*- or a 5-*O*-derivative. Galloylshikimic acid (compound **7**, *t*_R_ = 4.55 min) at 325 *m*/*z* showed a characteristic MS^2^ base peak fragment at 169 *m*/*z* and secondary MS^2^ peak at 125 *m*/*z* generated by a further loss of CO_2_. Compound **10** at 5.04 min, with a molecular ion at 495 *m*/*z* and MS^2^ base peak at 343 *m*/*z* (obtained by elimination of one galloyl group, 152 Da), was tentatively identified as digalloylquinic acid. It produced the MS^3^ base peak at 191 *m*/*z* and secondary MS^2^ peak at 169 *m*/*z*, which corresponded to the deprotonated quinic and gallic acids, respectively. Very similar fragmentation was also found for trigalloylquinic acid (compound **19**).

#### 2.1.2. Flavonoids and Their Derivatives

Considering the identified flavonoid glycosides, almost all of them (except naringin) were found to be 3- or 7-*O* derivatives of flavonols (kaempferol, quercetin, and myricetin) based on their MS fragmentation data (intensity of main ions in MS^2^, MS^3^, and MS^4^ spectra) presented in Table 1. It is well known that advanced LC-MS analysis may provide information regarding the structure of the aglycone and glycosidic part, as well as interglycosidic linkage [28]. Therefore, compound **42,** with a quasimolecular ion at 599 *m*/*z* and retention time at 6.98 min, was identified as kaempferol 3-*O*-(6″-galloyl)hexoside. It produced the MS^2^ base peak at 313 *m*/*z* (loss of kaempferol; 286 Da) and secondary MS^2^ peaks at 447 and 285 *m*/*z*, which were obtained by loss of the galloyl and galloylhexose unit, respectively. In the MS^3^ spectrum of compound **42**, we observed base peaks at 169 *m*/*z* (deprotonated gallic acid) and secondary MS^3^ peaks at 271, 241, and 211 *m*/*z*, which occurred during cross-ring cleavages of the sugar moiety. The proposed fragmentation pathway of compound **42** is depicted in Figure S1. Quercetin 3-*O*-(6″-*p*-hydroxybenzoyl)hexoside (compound **50**, *t*_R_ = 7.43 min) with a molecular ion at 583 *m*/*z* was identified in all of the investigated *A. unedo* leaf samples. It produced a characteristic MS^2^ base peak fragment at 463 *m*/*z* ([M–hydroxybenzoyl]^−^) and secondary MS^2^ peaks at 433 *m*/*z* (further elimination of 30 Da by cross-ring cleavage of hexose) and 301 *m*/*z* (deprotonated quercetin). Fragmentation of 301 *m*/*z* (MS^4^ spectra) confirmed the presence of quercetin as an aglycone.

#### 2.1.3. Other Phenolic Compounds

As for the other phenolic compounds, which were not phenolic acids or flavonoids, four of them (arbutin, aesculin, vanillin, and coniferyl aldehyde) were identified using available analytical standards.

Arbutin (compound **59**) and its derivative, galloylarbutin (compound **61**), which are known to be present in *A. unedo* in large quantities [8,29], were confirmed in all of the investigated samples. Digalloylarbutin (compound **62**) was identified only in leaf samples, and as far as we know, it has not been identified in *A. unedo* until now. In the MS^2^ spectra of galloylarbutin (compound **61**) at retention time 5.30 min and molecular ion 423 *m/z,* we observed the MS^2^ base peak at 313 *m*/*z* (loss of hydroquinone—110 Da) and secondary MS^2^ peak at 169 *m*/*z*. Further fragmentation of 169 *m*/*z* (formation of 125 *m*/*z*—loss of CO_2_) confirmed the presence of gallic acid. A similar fragmentation pathway was found for the other arbutin derivative, digalloylarbutin (compound **62**).

### 2.2. Quantitative Phenolic Profile of A. unedo Leaves and Fruit

Twenty phenolic compounds in total were quantified in all *A. unedo* extracts; data are shown in Table 2. As for phenolic acids, gallic acid was quantified only in fruit samples in the range of 19.30 mg/kg dw (Mali Lošinj, methanol extract) to 25.80 mg/kg dw (Koločep, MeOH extract). As previously reported in the literature [14,22,30], gallic acid was the most predominant phenolic acid in fruits. Protocatechuic acid, aesculin, chlorogenic acid, syringic acid, *p*-coumaric acid, ferulic acid, myricetin, naringenin, and kaempferol were not quantified in fruit samples. Flavan-3-ols were the dominant studied phenolics in fruits, with gallocatechin (74.63–91.08 mg/kg dw) and catechin (20.64–61.70 mg/kg dw) being the most abundant. These findings were in accordance with literature data [7,15,16,20]. Catechin gallate was found in only one *A. unedo* fruit sample from Mali Lošinj (methanol extract), while quercetin was also quantified in one fruit sample (from the same location, but in water extract).

According to the obtained results, flavan-3-ols (gallocatechin, catechin, and catechin gallate), hyperoside (quercetin 3-*O*-galactoside), and rutin (quercetin 3-*O*-rutinoside) were found in significant quantities in the investigated leaf extracts. The most abundant compound from the flavan-3-ol group was gallocatechin, ranging from 64.21 to 211.60 mg/kg dw for leaf samples. Gallocatechin and catechin were found in the highest quantities in the methanol leaf extract from Koločep (211.60 mg/kg dw and 102.95 mg/kg dw, respectively), while catechin gallate was the most abundant in methanol leaf extract from Mali Lošinj (124.91 mg/kg dw). Flavonol glycosides were the most abundant phenolic compounds found in leaves, which was in accordance with the literature data [9,13]. Among all of the quantified flavonol glycosides, hyperoside (quercetin 3-*O*-galactoside) was the most abundant (635.10–1512.94 mg/kg dw). It is worth mentioning that, according to the study by Maleš et al. [9], the concentrations of flavonol glycosides quercitrin and hyperoside, as well as chlorogenic acid in strawberry tree leaf samples collected on Pelješac and Dugi Otok, varied depending on the season of the year. Higher concentrations of quercitrin and hyperoside were found in January, and chlorogenic acid in the summer and early autumn. Of the nine phenolic acids quantified in our study, *p*-coumaric (10.11–32.83 mg/kg dw) and *p*-hydroxybenzoic acid (16.21–27.08 mg/kg dw) were the most abundant.

According to some authors, flavonol glycosides such as astragalin, hyperoside, myricitrin, quercitrin, and rutin, as well as phenolic acids (cinnamic, ferrulic, caffeic) and their esters catechine and arbutin, could be responsible for the strong antimicrobial activity of different parts of *A. unedo* [4,31,32]. Gallic acid and its derivatives identified in fruits have antioxidative, antifungal, antiherpetic, anti-infammatory, and anticancer activity [33,34], showing selective toxicity for cancer cells and lesser toxicity for normal healthy cells [35]. Gallic acid induces apoptosis in some tumor cell lines, and has an important role in preventing malignant transformation and cancer development in vivo [34]. The most dominant compounds that could be found in *A. unedo* leaf and fruit extracts were gallic acid derivatives, such as galloylquinic acids, galloylshikimic acids, gallic acid glycosides, glycosides of ellagic acid, and some galloyl derivatives of flavonols and flavan-3-ol [10,17,27].

#### Principal Component Analysis

Based on the content of the quantified phenolic compounds (Table 2) in the samples of *A. unedo* leaves and fruits, principal component analysis (PCA) was performed. The PCA was carried out at the exploratory level; therefore, it was not used as a classification model, but rather as a hint of what could be expected from the current data, and to check if there were some logical patterns in the data that might be explained.

**Figure 2:**
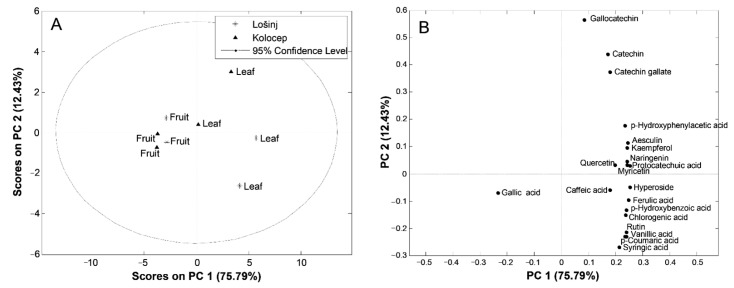
Principal component analysis (PCA): (**A**) score plot and (**B**) loading plot of the samples of *Arbutus unedo* leaves and fruits.

The principal component analysis resulted in a three-component model that explained 93.38% of the total variance. The first principal component, PC1, accounted for 75.79% of the overall data variance; the second one, PC2, for 12.43%; and the third principal component, PC3, for 5.16%. It is not unusual to obtain such high overall data variance captured by a few PCs, especially when the number of samples is small, the variability among the samples is relatively high (naturally occurring objects, samples), and a diverse set of parameters (variables) is considered. Mutual projections of factor scores and their loadings for the first two PCs are presented in Figure 2.

Taking into account the PC1 and PC2 score values (Figure 2A), two distinctive groups of samples were obtained. One of them belonged to samples of leaves, and the other one to samples of fruits. Fruit samples were widespread alongside the left part of the score plot. These samples were firmly clustered and distinguished with regard to the leaf samples. The score plot also revealed the existence of two well-separated groups between leaves corresponding to the different localities—Mali Lošinj and Koločep—where *A. unedo* leaves were randomly collected. The leaves collected on the island of Mali Lošinj formed a group at the lower right part of the plot, while the leaf samples collected on the island of Koločep were grouped in the upper right part (Figure 2A). The loading plot (Figure 2B) revealed that the most influential parameters discriminating the leaf samples from the samples of fruits were phenolic compounds **2**–**20** (Table 2), whose concentration was higher in the leaf samples, with the exception of gallic acid, which was present only in the fruit samples. Furthermore, the difference between the locality of leaf samples was determined by the content of gallocatechin, catechin, catechin gallate, and *p*-hydroxyphenilacetic acid (Figure 2B), whose content was higher in leaves from Koločep than the leaf samples from Mali Lošinj (Table 2), while the content of phenolic compounds **3**–**5**, **7**–**11**, **13**, **14**, and **16**–**20** was higher in leaf extracts from Mali Lošinj (Table 2). Discrimination of the samples on the basis of plant tissue and their geographical area confirmed data obtained in study of Maldini at al. [13], which was carried out on fruits and leaves collected at two locations in Sardinia.

### 2.3. Determination of TPC and RSA

Plant phenolics in general are highly effective free radical scavengers and antioxidants. Different types of phenolic extraction from leaves and fruits show different antioxidative capacities of extracts that depend not only on the extract composition, but also on the type of solvent used. Oliveira et al. [36] showed that extraction yields were higher for the water and methanol extracts of *A. unedo* leaves compared with ethanol and diethyl ether extracts, while Bouzid et al. [37] measured a notably higher amount of phenolics in chloroform, butanol, and ethyl acetate fruit extracts than in the aqueous one. Our results showed that methanol was a generally more effective solvent for phenolic extraction than water (Table 3).

The TPC and RSA in this study were slightly higher in leaves from Koločep than Mali Lošinj, and were in accordance with results from Portugal [10,24,36]. Higher TPC and RSA in leaves from Koločep could be explained by higher UV solar radiation and water deficit compared to Mali Lošinj [1]. Similarly, the higher phenolic content in samples from Algeria could be attributed to the different climate and location [38]. The RSA values found in our study were remarkably higher when compared with the RSA determined in leaf samples from Turkey, while the TPC values were in agreement [39]. The content of the bioactive compounds in *A. unedo* was strongly related to the conditions for its cultivation (climate, type/characteristics of the soil, soil management, harvesting time, etc.) [23,25,40,41,42].

Sonication during extraction was probably largely responsible for the higher TPC and stronger RSA in fruits from Mali Lošinj and Koločep than those determined in fruits from Portugal and Turkey [10,43,44,45]. To be more precise, ultrasound waves facilitate releasing phenolics from the complex matrix such as fruit [46]. The significantly higher TPC and RSA in Moroccan fruits could have been due to the environmental characteristics or fruit maturity at the time of harvesting [19]. The significantly higher antioxidant potential of Italian fruits compared to our results pointed to ethanol as the most promising extraction solvent for bioactive compounds in fruits [23]. This was further confirmed in a study by Zitouni et al. [20], who obtained a 50% higher TPC than we did using methanol for the extraction of phenolics from Moroccan fruits.

Both the TPC and RSA values were found to be remarkably higher in leaves than in fruits. As already reported [23,47], the antioxidant activity of phenolic compounds is related to the number of hydroxyl groups present in their structure, while the presence of a second hydroxyl group in the ortho position increases the antioxidant activity due to an additional resonance stabilization and formation of *o*-quinone. These characteristics can be used to explain the much stronger RSA in leaves than in fruits, as rutin and hyperoside, quantified in large amounts in leaves, possess large number of hydroxyl groups. It is also known that the most effective radical scavengers are flavonoids with the hydroxyl group at the C-3 position and/or 3′,4′-dihydroxy substitution on the B-ring [47]. Such flavonoids (e.g., quercetin, kaempferol, and rutin) were detected in a large quantity in leaves, while kaempferol was not detected in the fruits at all.

A strong correlation between TPC and DPPH (r = 0.929 and −0.976 for RSA measured as TE and EC50, respectively) indicated that phenolic compounds were responsible for the radical scavenging properties of *A. unedo* leaves and fruits. This correlation between the phenolic composition of extracts and their respective antioxidant activity has already been reported previously [10,12,13,23,36].

### 2.4. Strengths and Limitations of the Study

Although phenolic compounds in *A. unedo* leaves and fruits were determined in previous studies, this is the most comprehensive study on phenolics in *A. unedo* up to now, and the first report of 16 new phenolics in *A. unedo* leaf or fruit samples. In addition, the relation between antioxidant activity and structure of quantified phenolics in *A. unedo* was established for the first time. PCA showed that UHPLC-LTQ Orbitrap MS could be used to identify which phenolics were able to discriminate samples regarding plant tissue and geographical origin. However, in future studies, it will be necessary to collect more samples through different years from different geographical areas, to provide more data and increase knowledge on effects of climate, type/characteristics of the soil, harvesting time, etc., on the content of bioactive compounds in the *A. unedo* plant.

## 3. Materials and Methods

### 3.1. Chemicals and Materials

Acetonitrile and acetic acid (both MS grade) and methanol (HPLC grade) were purchased from Merck (Darmstadt, Germany). The 2,2-Diphenyl-1-picrylhydrazyl radical (DPPH^•^) (D9132) and phenolic standards were supplied by Sigma-Aldrich (Steinheim, Germany). Trolox ((±)-6-hydroxy-2,5,7,8-tetramethyl-chromane-2-carboxylic acid) (56510) was purchased from Fluka (Buchs, Germany). Sodium carbonate and Folin–Ciocalteu reagent were obtained from Kemika (Zagreb, Croatia).

To prepare the standard solutions and blanks, ultra-pure water (Thermo Fisher TKA MicroPure water purification system, 0.055 µS/cm, Niederelbert, Germany) was used. Syringe filters (13 mm, PTFE membrane 0.45 µm) were supplied by Supelco (Bellefonte, PA, USA).

### 3.2. Samples

*A. unedo* leaves were randomly collected on the Croatian islands of Mali Lošinj (GPS coordinates: 44°31′50″ N; 14°28′06″ E; 14 m a.s.l., north Adriatic) and Koločep (GPS coordinates: 42°40′34″ N; 18°00′35″ E; 34 m a.s.l., south Adriatic) in October 2013. Dario Kremer (Faculty of Pharmacy and Biochemistry, University of Zagreb, Croatia) identified the herbal material. The voucher specimen (No. IMI-HR 0046/2013) was deposited at the Institute for Medical Research and Occupational Health, Zagreb, Croatia. The leaves were air-dried in a dark ventilated room at 22 °C for 20 days. The dried leaves were grounded by a laboratory mill (A 10 basic, IKA, Staufen, Germany) and stored at room temperature in the dark and in plastic containers [29,48]. The fresh leaves of both samples were weighed (Mettler AE 200, Mettler-Toledo, Columbus, OH, USA), dried to constant weight, and weighed again after 20 days. The leaf moisture content was calculated as follows: moisture content (%) = (fw − dw)/fw × 100, where fw is fresh leaf weight and dw is dried leaf weight. Each sample was measured in triplicate. Moisture content in leaves from Mali Lošinj and Koločep, shown as mean ± standard deviation, was 78.1 ± 3.4 and 75.4 ± 2.1, respectively.

Fruits of *A. unedo* in red mature stage, uniform in shape and colour, were collected at the same time and location as the leaves. Fruits were immediately frozen at −20 °C, lyophilized in a HETOSIC lyophiliser (HETO, Denmark), and stored in a desiccator until analysis.

### 3.3. Preparation of the Extracts

To prepare extracts, 3 g of dried powdered leaves or lyophilised fruits were extracted with 80 mL of methanol or water in an ultrasound bath (Bandelin Sonorex, Berlin, Germany) at 50 °C for 60 min. Samples were vortexed (Vortex 3, IKA, Staufen, Germany) every 15 min. The extracts were filtered through a 0.45 µm PTFE membrane filter (Supelco, Bellefonte, PA, USA) after cooling to room temperature. Obtained extract aliquots were used to determine the total phenolic content and antiradical activity.

To determine the phenolic profile by UHPLC-LTQ OrbiTrap MS, aliquots of water and methanolic extracts were lyophilised and dissolved in ultrapure water for analysis.

### 3.4. UHPLC-LTQ OrbiTrap MS Analysis of Phenolic Compounds

To broaden knowledge regarding phenolic content, we used ultra-high performance liquid chromatography (UHPLC) coupled with a hybrid mass spectrometer (LTQ Orbitrap MS). The application of this hybrid technique enabled a simultaneous and unambiguous detection of a large number of phenolic compounds based on the high-resolution, accurate mass measurement and fragmentation pattern [49].

A 1000 mg/L stock solution of a mixture of all phenolic standards was prepared in methanol. The dilution of the stock solution with methanol yielded the working solutions in concentrations of 0.025, 0.050, 0.100, 0.250, 0.500, 0.750, and 1.000 mg/L. Calibration curves were obtained by plotting the peak areas of the standards against their concentration. For the analysis, 5 mg of lyophilisate was dissolved in 3 mL of ultrapure water. The injection volume was 5 μL.

Chromatographic separations were performed using an ultra-high performance liquid chromatography (UHPLC) system consisting of a quaternary Accela 600 pump and Accela autosampler (Thermo Fisher Scientific, Bremen, Germany). The UHPLC system was coupled to a linear ion trap-OrbiTrap hybrid mass spectrometer (LTQ OrbiTrap MS) equipped with a heated electrospray ionisation probe (HESI-II, Thermo Fisher Scientific). A Syncronis C18 column (100 × 2.1 mm, 1.7 µm particle size) from Thermo Fisher Scientific was used as the analytical column for separation. The mobile phase consisted of (A) water + 0.01% acetic acid and (B) acetonitrile. A linear gradient program at a flow rate of 0.250 mL/min was used: 0.0–1.0 min 2% B, 1.0–14.0 min from 2% to 98% (B), 14.0–14.1 min from 98% to 2% (B), then 2% (B) for 5 min.

The mass spectrometer was operated in negative mode. Parameters of the ion source were as follows: source voltage 4.5 kV, capillary voltage –4 V, tube lens voltage –59.11 V, capillary temperature 275 °C, sheath and auxiliary gas flow (N_2_) 30 and 7 (arbitrary units). The MS spectra were acquired by full-range acquisition covering 100–1000 *m*/*z*. Resolution was set at 30,000 for full scan analysis. The data-dependent MS/MS events were always performed on the most intense ions detected in the full scan MS. The ions of interest were isolated in the ion trap with an isolation width of 5 ppm and activated with 35% collision energy levels (CEL). The dynamic exclusion was used with the following settings: repeat count 1; repeat duration 4 s; exclusion list size 500; exclusion duration 10 s. Full scan analysis was employed to calculate the monoisotopic mass of unknown compounds, while the fragmentation pathway was obtained by MS*^n^* and confirmed using Mass Frontier 6.0 software (Thermo Fisher Scientific). The molecule editor program ChemDraw (version 12.0) was used to calculate an accurate mass of the compounds of interest. The tentative identification of compounds for which standards were not available was achieved using previously reported MS fragmentation data found in the literature.

Phenolics were identified and quantified according to the corresponding spectral characteristics: mass spectra, accurate mass, characteristic fragmentation, and characteristic retention time. Table S1 presents the list of quantified phenolics in the investigated samples with regression equation parameters, correlation coefficients, and limits of detection (LOD) and quantification (LOQ) obtained from the calibration curves created in MS Excel. Xcalibur software (version 2.1) was used for instrument control, data acquisition and data analysis. The total amounts of each compound were evaluated by calculation of the peak areas and expressed as mg/kg dried weight (dw).

### 3.5. Total Phenolic Content (TPC)

Determination of total phenolics was performed with a slightly modified Folin–Ciocalteu’s spectrophotometric assay described by Jurica et al. [48]. A total of 50 µL of solvent-diluted sample extract (1:20 and 1:5 (*v*/*v*) for leaf and fruit extract, respectively) was mixed with 1.42 mL of water and 80 µL of Folin–Ciocalteu reagent. After 5 min, 1.5 mL of 6% sodium carbonate solution (*w*/*v*) was added. The obtained solution was incubated at 40 °C for 30 min (Thermostat Instrumentaria, Zagreb, Croatia) to allow the development of the characteristic blue colour and then cooled to room temperature, after which the absorbance was measured at 765 nm (Cary 50, Varian, Mulgrave, Australia). TPC was determined from a standard curve of gallic acid standard solution (5–200 mg/L).The results were expressed in mg of gallic acid equivalents (GAE) per g of dried leaf/fruit weight.

### 3.6. DPPH Radical Scavenging Activity (RSA)

For the evaluation of radical scavenging activity two different methods were used.

First, the DPPH free radical scavenging capacity of leaf sample extracts was determined as described by Jurica et al. [48]. Briefly, five dilution levels of the strawberry tree leaf extracts (1:500–1:4000, *v*/*v*) and strawberry tree fruit extracts (1:125–1:1000, *v*/*v*) were prepared for each sample using methanol for dilution. Then, 2 mL of leaf or fruit extract was mixed with 1.5 mL of DPPH and incubated at room temperature for 30 min. The percentage of absorbance reduction after 30 min measured at 528 nm (Cary 50, Varian, Mulgrave, Australia) was plotted against the concentration of a measured extract, and the calibration curve was constructed for each sample. The effective extract concentration (EC_50_), which caused a 50% decrease of the initial concentration of the DPPH radical, was calculated from the graph of % radical scavenging activity (RSA) plotted against the extract concentration (mg/L).

Secondly, the DPPH radical scavenging activity method was also performed by using Trolox reagent. An aliquot (0.1 mL) of diluted leaf and fruit extract was mixed with 0.9 mL of methanol. The sample solution was then mixed with 1.5 mL of DPPH methanolic solution (0.18 mmol/L) and vortexed vigorously. The absorbance was measured at 517 nm after incubation in the dark for 30 min at 25 °C. A calibration curve in the range 0.01–0.1 mmol/L was used for the Trolox, and results were expressed as micromoles of Trolox equivalent antioxidant capacity per g of dried leaf/fruit weight (μmol TE/g).

### 3.7. Statistical Analysis

Statistical analyses were performed using Statistica 13 for Windows (StatSoft Inc., Tulsa, OK, USA). Spectrophotometric measurements were carried out in triplicate, and results were expressed as the mean ± standard deviation (SD). Correlation analysis between TPC and DPPH was performed by Spearman correlation, as the data were not normally distributed.

Principal component analysis (PCA) was performed using PLS ToolBox, v.6.2.1, for MATLAB 7.12.0 (R2011a). All data were pretreated (autoscaled) before statistical operations in order to prevent highly abundant components to dominate in the final result. PCA was carried out by using a singular value decomposition algorithm and a 0.95 confidence level for Q and T2 Hotelling limits for outliers. Using only a limited number of principal components (PCs), the dimensionality of the retention data space was educed, further analysis was simplified, and the parameters were grouped according to similarities.

## 4. Conclusions

This study reported the investigation of the main phytochemicals found in strawberry tree (*A. unedo* L., Ericaceae) leaves and fruits originating from Croatia. The phytochemicals found in this plant-derived food have a protective role in human health, acting primarily as antioxidants. Generally, *A. unedo* leaf and fruit extracts showed a strong radical scavenging effect. Techniques that combined chromatographic with high-resolution and high mass accuracy spectral methods, such as the UHPLC-LTQ OrbiTrap MS technique, were proven to be very useful in obtaining information on the chemical structures of some compounds with high reliability. Using this technique, a total of 64 different compounds, including phenolic acids and their derivatives, flavonoid aglycones and glycosides, as well as some arbutin derivatives, were identified in *A. unedo* leaves and fruits. The most abundant compounds were found to be derivatives of gallic acid. Among all of the identified compounds, 20 were quantified using available standards, while the other compounds were identified on the basis of their deprotonated molecule combined with its MS^2^, MS^3^, and MS^4^ fragmentation patterns. Hyperoside and flavan-3-ols were the predominant compounds in leaf samples, while gallocatechin and catechin were the major compounds found in fruit samples. Some of identified compounds were detected in trace amounts in the tested samples and proven using available standards, while others were tentatively identified using high-resolution and multistage mass spectrometry. This study also highlighted the fact that the antioxidant activity of the studied compounds was strongly correlated with their chemical structure.

The established phytochemical profiles of *A. unedo* leaves and fruits could provide valuable information about the potential health-promoting effects of this plant, and on the other hand could influence the positioning of strawberry tree products on the Croatian and world food market. This wild Mediterranean plant has good potential to be a part of organized agricultural cultivation with food safety standards implemented in cultivation and technological processing and effective food authenticity control systems, leading to prospective maximum utilization of *A. unedo* fruits and leaves in the food industry, or in phytopharmaceutics, to be used as a complement to traditional therapeutics in the prevention and alleviation of diseases.

## Figures and Tables

**Table 1:** Phytochemical fingerprint of strawberry tree (*Arbutus unedo* L., Ericaceae) leaves and fruits from Croatia using UHPLC-LTQ Orbitrap MS.

No.	Compound Name ^a^	*t*_R_ (min)	Molecular Formula [M–H]^–^	Calculated Mass [M–H]^–^	Exact Mass [M–H]^–^	Δ ppm	MS^2^ Fragments (% Base Peak)	MS^3^ Fragments (% Base Peak)	MS^4^ Fragments (% Base Peak)
*Phenolic acids and their derivatives*									
**1**	**Gallic acid** ^F^	3.13	C_7_H_5_O_5_^−^	169.01425	169.01350	4.44	**125**(100)	**107**(100)	-
**2**	Galloylquinic acid ^L,F^	3.21	C_14_H_15_O_10_^−^	343.06707	343.06540	4.87	**191**(100), 169(10)	173(80), **127**(100), 85(90)	**85**(100)
**3**	Hydroxybenzoic acid hexoside isomer 1 ^L,F^	4.03	C_13_H_15_O_8_^−^	299.07724	299.07579	4.85	**137**(100)	**93**(100)	-
**4**	Dihydroxybenzoic acid hexoside ^L,F^	4.10	C_13_H_15_O_9_^−^	315.07216	315.07065	4.79	**153**(100), 152(50), 109(15), 108(10)	**109**(100)	**84**(100), 81(60)
**5**	Gallic acid dihexoside ^L^	4.15	C_19_H_25_O_15_^−^	493.11989	493.11829	3.24	**433**(100), 331(20), 313(80), 283(30), 169(20)	**323**(100), 161(10)	179(20), **161**(100), 143(20)
**6**	Gallic acid hexoside ^L,F^	4.31	C_13_H_15_O_10_^−^	331.06707	331.06540	5.04	**169**(100), 125(5)	**125**(100)	**107**(100), 83(10), 65(5)
**7**	Galloylshikimic acid ^L,F^	4.55	C_14_H_13_O_9_^−^	325.05651	325.05497	4.74	**169**(100), 125(15)	**125**(100)	**107**(100), 97(50), 81(40), 79(10), 69(5)
**8**	**Protocatechuic acid** ^L^	4.68	C_7_H_5_O_4_^−^	153.01933	153.01868	4.25	**109**(100), 107(20), 95(5)	81(50), **79**(100)	-
**9**	Hydroxybenzoic acid hexoside isomer 2 ^L,F^	4.70	C_13_H_15_O_8_^−^	299.07724	299.07590	4.48	**137**(100)	**93**(100)	-
**10**	Digalloylquinic acid ^L,F^	5.04	C_21_H_19_O_14_^−^	495.07803	495.07611	3.88	**343**(100), 325(10), 301(10), 191(15), 169(5)	**191**(100), 169(20)	173(70), 127(90), **85**(100)
**11**	Digalloylshikimic acid ^L,F^	5.22	C_21_H_17_O_13_^−^	477.06746	477.06525	4.63	417(10), **325**(100)	**169**(100), 125(15)	**125**(100)
**12**	Coumaric acid hexoside ^L^	5.35	C_15_H_17_O_8_^−^	325.09289	325.09164	3.85	**163**(100), 119(10)	**119**(100)	-
**13**	**5-*O*-Caffeoylquinic acid (Chlorogenic acid)** ^L^	5.38	C_16_H_17_O_9_^−^	353.08781	353.08688	2.63	**191**(100), 179(5)	173(75), **127**(100), 111(40), 93(60), 85(90)	109(30), 99(60), **85**(100)
**14**	***p*- Hydroxybenzoic acid** ^L,F^	5.51	C_7_H_5_O_3_^−^	137.02442	137.02390	3.79	109(10), **93**(100)	**93**(100)	-
**15**	**Caffeic acid** ^L,F^	5.73	C_9_H_7_O_4_^−^	179.03498	179.03413	4.75	**135**(100), 119(15), 117(10), 91(20), 59(15)	107(30), **91**(100), 89(80), 59(10)	-
**16**	Ellagic acid hexoside ^L,F^	5.84	C_20_H_15_O_13_^−^	463.05181	463.05075	2.29	302(15), **301**(100), 300(50), 289(10), 273(10)	301(70), 284(20), **257**(100), 229(65), 185(30)	229(50), 213(30), **185**(100)
**17**	**Syringic acid** ^L,F^	5.85	C_9_H_9_O_5_^−^	197.04555	197.04462	4,72	**182**(100), 153(50), 138(10)	**167**(100), 138(10), 123(5)	**123**(100)
**18**	**Vanillic acid** ^L,F^	5.91	C_8_H_7_O_4_^−^	167.03498	167.03444	3.23	153(10), 152(80), 124(10), **123**(100), 108(20)	**108**(100)	123(30), 80(35), **78**(100)
**19**	Trigalloylquinic acid ^L^	5.93	C_28_H_23_O_18_^−^	647.08899	647.08722	2.74	**495**(100), 343(5)	**343**(100), 325(5), 191(15)	**191**(100), 169(15)
**20**	Trigalloylshikimic acid ^L^	6.22	C_28_H_21_O_17_^−^	629.07842	629.07654	2.99	**477**(100), 325(5)	**325**(100)	**169**(100), 125(15)
**21**	Ellagic acid pentoside ^L,F^	6.30	C_19_H_13_O_12_^−^	433.04125	433.04037	2.03	302(15), **301**(100), 300(60)	301(60), 300(40), 284(25), **257**(100), 229(70)	240(10), **229**(100), 213(30), 201(20), 185(90)
**22**	***p*-Hydroxyphenylacetic acid** ^L,F^	6.57	C_8_H_7_O_3_^−^	151.04007	151.03957	3.31	**136**(100), 95(5)	108(25), **92**(100)	**108**(100)
**23**	***p*-Coumaric acid** ^L,F^	6.69	C_9_H_7_O_3_^−^	163.04007	163.03944	3.86	149(40), **119**(100)	104(60), **77**(100)	-
**24**	**Ellagic acid** ^L,F^	6.77	C_14_H_5_O_8_^−^	300.99899	300.99750	4.95	284(40), 271(60), **257**(100), 229(85), 185(40)	**229**(100), 213(20), 185(85)	**201**(100), 185(95), 157(30), 145(20), 129(10)
**25**	**Ferulic acid** ^L,F^	6.88	C_10_H_9_O_4_^−^	193.05063	193.04982	4.20	178(70), **149**(100), 134(50)	**134**(100)	**106**(100)
**26**	**Cinnamic acid** ^L^	8.84	C_9_H_7_O_2_^−^	147.04515	147.04451	4.35	104(10), **103**(100), 87(10)	**119**(100)	-
*Flavonoids and their derivatives*									
**27**	**Gallocatechin** ^L,F^	4.63	C_15_H_13_O_7_^−^	305.06668	305.06537	4.29	261(50), 221(70), 219(70), **179**(100), 165(35)	**164**(100), 151(40), 135(30)	**120**(100), 108(20)
**28**	**Catechin** ^L,F^	5.50	C_15_H_13_O_6_^−^	289.07176	289.07053	4.25	271(5), **245**(100), 205(40), 179(15), 125(5)	227(30), **203**(100), 187(25), 175(10), 161(20)	188(70), 185(20), **175**(100), 161(40), 157(10)
**29**	Myricetin 3-*O*-(6″-rhamnosyl)glucoside ^L^	6.09	C_27_H_29_O_17_^−^	625.14102	625.13920	2.91	607(15), 317(80), **316**(100), 287(5), 271(15)	287(40), **271**(100), 179(30)	271(15), **243**(100)
**30**	Myricetin 3-*O*-hexoside ^L,F^	6.24	C_21_H_19_O_13_^−^	479.08311	479.08087	4.68	463(30), 455(10), 317(80), **316**(100)	287(30), **271**(100), 179(40)	271(15), **243**(100), 227(30)
**31**	Myricetin 3-*O*-pentoside ^L,F^	6.40	C_20_H_17_O_12_^−^	449.07255	449.07123	2.94	318(10), **317**(100), 316(80), 315(20)	272(25), **179**(100), 151(40)	**151**(100)
**32**	**Quercetin 3-*O*-(6″-rhamnosyl)glucoside (Rutin)** ^L,F^	6.48	C_27_H_29_O_16_^−^	609.14611	609.14392	3.60	343(5), **301**(100), 300(30), 271(10), 255(5)	273(25), 257(20), **179**(100), 151(75)	**151**(100)
**33**	Quercetin 3-*O*-(6″-galloyl)hexoside ^L,F^	6.49	C_28_H_23_O_16_^−^	615.09916	615.09662	4.13	**463**(100), 301(20)	343(5), **301**(100), 300(40)	273(15), 257(30), 229(20), **179**(100), 151(90)
**34**	Myricetin 7-*O*-pentoside ^L,F^	6.58	C_20_H_17_O_12_^−^	449.07255	449.07108	3.27	318(10), **317**(100)	272(25), **179**(100), 151(40)	**151**(100)
**35**	Myricetin 3-*O*-rhamnoside (Myricitrin) ^L^	6.65	C_21_H_19_O_12_^−^	463.08820	463.08661	3.43	317(50), **316**(100)	287(30), **271**(100), 179(40)	271(15), **243**(100), 227(30)
**36**	**Quercetin 3-*O*-galactoside (Hyperoside)** ^F^	6.68	C_21_H_19_O_12_^−^	463.08820	463.08685	2.92	**301**(100), 300(30)	273(25), 257(20), **179**(100), 151(75)	**151**(100)
**37**	**Catechin 3-gallate** ^L,F^	6.78	C_22_H_17_O_10_^−^	441.08272	441.08118	3.49	331(10), **289**(100), 271(10), 169(25)	271(5), **245**(100), 205(40), 179(20)	227(20), **203**(100), 187(20), 175(10), 161(20)
**38**	Kaempferol 7-*O*-(6″-rhamnosyl)glucoside ^L,F^	6.85	C_27_H_29_O_15_^−^	593.15119	593.14850	4.54	**285**(100)	267(50), **257**(100), 241(40), 229(50), 213(30)	255(20), 239(30), **229**(100), 213(30), 163(60)
**39**	Myricetin 7-O-hexuronide ^L^	6.87	C_21_H_17_O_14_^−^	493.06238	493.06064	3.53	**317**(100)	193(15), **179**(100), 151(45)	**151**(100)
**40**	**Morin** ^L^	6.91	C_15_H_9_O_7_^−^	301.03538	301.03403	4.48	**286**(100), 273(90), 257(15), 207(20)	285(10), **268**(100), 257(70), 240(40), 229(15)	**240**(100), 212(10)
**41**	Quercetin 3-*O*-pentoside isomer 1 ^L,F^	6.93	C_20_H_17_O_11_^−^	433.07763	433.07608	3.58	**301**(100), 300(15)	283(20), 273(25), 257(10), **179**(100), 151(75)	**151**(100)
**42**	Kaempferol 3-*O*-(6″-galloyl)hexoside ^L^	6.98	C_28_H_23_O_15_^−^	599.10424	599.10175	4.16	447(70), **313**(100), 285(50), 284(25), 271(10)	241(20), 211(10), **169**(100), 151(5), 125(15)	**125**(100)
**43**	**Naringin** ^L,F^	7.02	C_27_H_31_O_14_^−^	579.17193	579.16919	4.73	**459**(100), 357(5), 313(25), 271(45), 235(10)	441(30), **357**(100), 339(30), 271(55), 235(85)	**339**(100), 169(20), 151(50), 125(20)
**44**	**Kaempferol 3-*O*-glucoside (Astragalin)** ^F^	7.05	C_21_H_19_O_11_^−^	447.09329	447.09183	3.27	327(20), 285(80), **284**(100), 255(10)	**255**(100), 227(10)	**227**(100), 211(60)
**45**	Quercetin 3-*O*-pentoside isomer 2 ^L,F^	7.07	C_20_H_17_O_11_^−^	433.07763	433.07645	2.72	**301**(100), 300(15)	283(20), 273(25), 257(10), **179**(100), 151(75)	**151**(100)
**46**	Quercetin 3-*O*-rhamnoside (Quercitrin) ^F^	7.17	C_21_H_19_O_11_^−^	447.09329	447.09113	4.83	**301**(100), 300(35), 284(20)	273(25), 257(20), **179**(100), 151(75)	**151**(100)
**47**	**Myricetin** ^L^	7.26	C_15_H_9_O_8_^−^	317.03029	317.02930	3.12	299(10), 273(35), **207**(100),163(95)	**179**(100), 151(15)	**151**(100)
**48**	Kaempferol 3-*O*-pentoside isomer 1 ^L,F^	7.31	C_20_H_17_O_10_^−^	417.08272	417.08115	3.76	327(10), 285(30), **284**(100), 255(10)	**255**(100), 227(15)	255(15), **227**(100), 211(80), 167(15)
**49**	Quercetin 3-*O*-hexuronide ^L,F^	7.40	C_21_H_17_O_13_^−^	477.06746	477.06552	4.07	302(15), **301**(100), 299(10)	273(20), 257(15), **179**(100), 151(80)	**151**(100)
**50**	Quercetin 3-*O*-(6″-*p*-hydroxybenzoyl)hexoside ^L^	7.43	C_28_H_23_O_14_^−^	583.10933	583.10754	3.07	**463**(100), 433(10), 301(50), 300(25), 271(10)	**301**(100), 300(40)	273(15), 257(30), 229(20), **179**(100), 151(90)
**51**	Kaempferol 3-*O*-pentoside isomer 2 ^L^	7.53	C_20_H_17_O_10_^−^	417.08272	417.08142	3.12	327(5), **285**(100), 284(70), 255(5)	267(50), **257**(100), 241(40), 229(60), 213(30)	255(15), 239(40), **229**(100), 213(30), 163(60)
**52**	Kaempferol 7-*O*-hexuronide ^L^	7.58	C_21_H_17_O_12_^−^	461.07255	461.07108	3.19	**285**(100)	**285**(100), 284(60)	267(40), **257**(100), 229(50), 213(30), 163(20)
**53**	Kaempferol 3-*O*-rhamnoside ^L,F^	7.63	C_21_H_19_O_10_^−^	431.09837	431.09674	3.78	**285**(100), 284(20)	267(50), **257**(100), 241(40), 229(50), 213(30)	255(20), 239(30), **229**(100), 213(30), 163(60)
**54**	Quercetin 3-*O*-(6″-*p*-coumaroyl) hexoside ^L^	7.85	C_30_H_25_O_14_^−^	609.12498	609.12268	3.78	**463**(100), 301(20)	**301**(100), 300(25)	273(25), 257(20), **179**(100)
**55**	Kaempferol 3-*O*-(6″-*p*-coumaroyl)hexoside ^L^	8.29	C_30_H_25_O_13_^−^	593.13006	593.12769	4.00	447(15), 307(10), **285**(100)	**257**(100), 241(50), 229(35), 213(40), 151(90)	256(10), 239(25), **229**(100), 213(20), 163(35)
**56**	**Quercetin** ^L,F^	8.62	C_15_H_9_O_7_^−^	301.03538	301.03391	4.88	271(50), 255(20), **179**(100), 151(80), 107(5)	**151**(100)	**107**(100), 83(10)
**57**	**Naringenin** ^L^	9.35	C_15_H_11_O_5_^−^	271.06120	271.06030	3.32	225(5), 177(10), **151**(100)	**107**(100)	**65**(100)
**58**	**Kaempferol** ^L^	9.51	C_15_H_9_O_6_^−^	285.04046	285.03909	4.81	**255**(100), 227(10)	**211**(100), 195(5), 167(15)	211(40), **137**(100)
*Other phenolic compounds*									
**59**	**Arbutin** ^L,F^	2.52	C_12_H_15_O_7_^−^	271.08233	271.08148	3.14	193(5), **161**(100), 113(10), 109(35), 101(5)	143(10), 129(20), 113(50), **101**(100), 71(50)	-
**60**	**Aesculin** ^L^	5.06	C_15_H_15_O_9_^−^	339.07216	339.07114	3.01	**177**(100)	177(5), 149(10), **133**(100), 105(10), 89(5)	**89**(100)
**61**	Galloylarbutin ^L,F^	5.30	C_19_H_19_O_11_^−^	423.09329	423.09213	2.74	**313**(100), 169(45)	**169**(100), 125(25)	**125**(100)
**62**	Digalloylarbutin ^L^	6.04	C_26_H_23_O_15_^−^	575.10424	575.10211	3.70	**423**(100)	**313**(100), 261(95), 211(5), 16930), 151(25)	**169**(100), 151(5), 125(20)
**63**	**Vanillin** ^L,F^	6.83	C_8_H_7_O_3_^−^	151.04007	151.03960	3.11	**136**(100)	108(25), **92**(100)	108(65), 79(65), **69**(100), 51(30)
**64**	**Coniferyl aldehyde** ^L,F^	7.80	C_10_H_9_O_3_^−^	177.05572	177.05485	4.91	163(10), **162**(100)	**134**(100), 133(40), 120(20), 106(30)	**106**(100), 65(80)

**^a^Bolded** compounds were confirmed using available analytical standards, all of the other compounds were identified based on MS data. Underlined compounds were identified in *A. unedo* extract for the first time. L—compound found in *A. unedo* leaves; F—compound found in *A. unedo* fruits; t_R_—mean expected retention times; Δ ppm–mean mass accuracy.

**Table 2:** Quantification of some phenolic compounds in strawberry tree (*Arbutus unedo* L., Ericaceae) leaves and fruits.

Phenolic Compounds		Leaf (mg/kg dw)				Fruit (mg/kg dw)			
		Mali Lošinj		Koločep		Mali Lošinj		Koločep	
		Water	MeOH	Water	MeOH	Water	MeOH	Water	MeOH
**1**	Gallic_acid	-	-	-	-	21.04	19.30	23.95	25.80
**2**	Gallocatechin	64.21	97.77	121.11	211.60	86.08	90.25	74.63	91.08
**3**	Protocatechuic acid	1.68	2.47	1.27	1.69	-	-	-	-
**4**	Aesculin	2.56	5.88	1.95	3.97	-	-	-	-
**5**	Chlorogenic acid	1.95	1.59	-	1.11	-	-	-	-
**6**	Catechin	47.73	79.25	57.85	102.95	33.17	61.70	20.64	48.33
**7**	*p*-Hydroxybenzoic acid	27.08	22.17	16.76	16.21	5.85	2.35	0.68	4.93
**8**	Caffeic acid	4.31	5.75	2.61	4.07	4.83	2.68	2.55	0.41
**9**	Syringic acid	2.67	1.27	0.66	0.88	-	-	-	-
**10**	Vanillic acid	7.96	7.12	4.40	3.71	1.74	2.54	1.64	1.52
**11**	Rutin	93.39	106.03	29.93	33.75	2.29	2.93	0.88	1.94
**12**	*p*-Hydroxyphenylacetic acid	4.35	5.36	4.63	6.57	-	-	-	-
**13**	Hyperoside	1149.54	1512.94	635.10	876.61	37.06	47.90	13.86	23.73
**14**	*p*-Coumaric acid	32.83	31.38	10.11	11.41	-	-	-	-
**15**	Catechin gallate	34.48	73.70	40.67	97.14	-	63.73	-	-
**16**	Ferulic acid	4.85	4.26	2.55	3.08	-	-	-	-
**17**	Myricetin	1.36	1.78	-	1.49	-	-	-	-
**18**	Quercetin	61.80	124.91	41.28	82.44	89.74	-	-	-
**19**	Naringenin	3.70	4.39	-	4.18	-	-	-	-
**20**	Kaempferol	15.15	35.50	10.63	22.26	-	-	-	-

The contents of some compounds (ellagic acid, cinnamic acid, morin, naringin, astragalin, myricetin, arbutin, vanillin, and coniferyl aldehyde) were found in trace amounts, and therefore were not reported in this table.

**Table 3:** Total phenolic content (TPC) and radical scavenging activity (RSA) in strawberry tree (*Arbutus unedo* L.) leaf and fruit extracts.

Sample	Location	TPC		RSA			
		(mg GAE/g dw *)		EC_50_ ** (mg/L)		μmol TE/g dw *	
		Water	Methanol	Water	Methanol	Water	Methanol
**Leaf**	Mali Lošinj	67.07 ± 1.92	85.30 ± 1.81	62.53 ± 1.13	45.12 ± 0.98	408.92 ± 2.68	430.70 ± 2.11
	Koločep	91.56 ± 0.45	104.74 ± 1.67	57.94 ± 1.50	38.23 ± 1.47	430.98 ± 0.84	428.36 ± 2.19
**Fruit**	Mali Lošinj	18.73 ± 1.41	25.86 ± 0.72	265.67 ± 1.81	177.49 ± 1.18	90.55 ± 0.49	104.04 ± 0.80
	Koločep	16.78 ± 0.91	20.38 ± 0.36	294.85 ± 1.94	256.72 ± 2.50	74.30 ± 0.09	97.54 ± 0.88

The values shown are the mean ± standard deviation of three replications. * Results expressed on dried leaf/fruit mass weight (dw); ** leaf/fruit extract concentration (mg/mL) to produce 50% reduction of the DPPH; GAE—gallic acid equivalents; TE—Trolox equivalents.

## Data Availability

The data presented in this study are available on request from the corresponding authors.

## Supplementary Materials

The following are available online at https://www.mdpi.com/article/10.3390/plants11010025/s1, Figure S1: Proposed fragmentation pathway of compound **42** (kaempferol 3-*O*-(6″-galloyl)hexoside), Table S1: The list of phenolic compounds with regression equation parameters, correlation coefficients, and limits of detection (LOD) and quantification (LOQ), obtained from the calibration curves.
