# Supplementary material for: Comparative Study on the Phenolic Fingerprint and Antioxidant Activity of Strawberry Tree (Arbutus unedo L.) Leaves and Fruits
Source: Plants (Basel). 2021 Dec 22;11(1):25. doi: 10.3390/plants11010025 (PMC8747429; doi:10.3390/plants11010025)
Supplement: Supplementary file 1 [file plants-11-00025-s001.zip › plants-1510210-supplementary.pdf]

Supplementary material

Article

Comparative Study on the Phenolic Fingerprint and Antioxidant Activity of Strawberry Tree (*Arbutus unedo* L.) Leaves and Fruits

Irena Brčić Karačonji [1,2,*,#], Karlo Jurica [3,#], Uroš Gašić [4,*], Aleksandra Dramićanin [5], Živoslav Tešić [5] and Dušanka Milojković Opsenica [5]

[1] Analytical Toxicology and Mineral Metabolism Unit, Institute for Medical Research and Occupational Health, Ksaverska cesta 2, 10000 Zagreb, Croatia; ibrcic@imi.hr (I.B.K.)
[2] Faculty of Health Studies, University of Rijeka, Viktora Cara Emina 5, 51000 Rijeka, Croatia
[3] Special Security Operations Directorate, Ministry of the Interior, Ulica grada Vukovara 33, 10000 Zagreb, Croatia; juricakarlo@gmail.com (K.J.)
[4] Institute for Biological Research "Siniša Stanković"–National Institute of Republic of Serbia, University of Belgrade, Bulevar despota Stefana 142, 11060 Belgrade, Serbia; uros.gasic@ibiss.bg.ac.rs (U.G.)
[5] Faculty of Chemistry, University of Belgrade, Studentski trg 12-16, 11158 Belgrade, Serbia; akosovic@chem.bg.ac.rs (A.D.); ztesic@chem.bg.ac.rs (Ž.T.); dusankam@chem.bg.ac.rs (D.M.O.)

* Correspondence: ibrcic@imi.hr (I.B.K.); uros.gasic@ibiss.bg.ac.rs (U.G.)
[#] Equally contributed (I.B.K. and K.J.)

Figure S1. Proposed fragmentation pathway of compound 42 (kaempferol 3-*O*-(6''-galloyl)hexoside).

Table S1. The list of phenolic compounds with regression equation parameters, correlation coeffi-cients, limits of detection (LOD) and quantification (LOQ), obtained from the calibration curves.

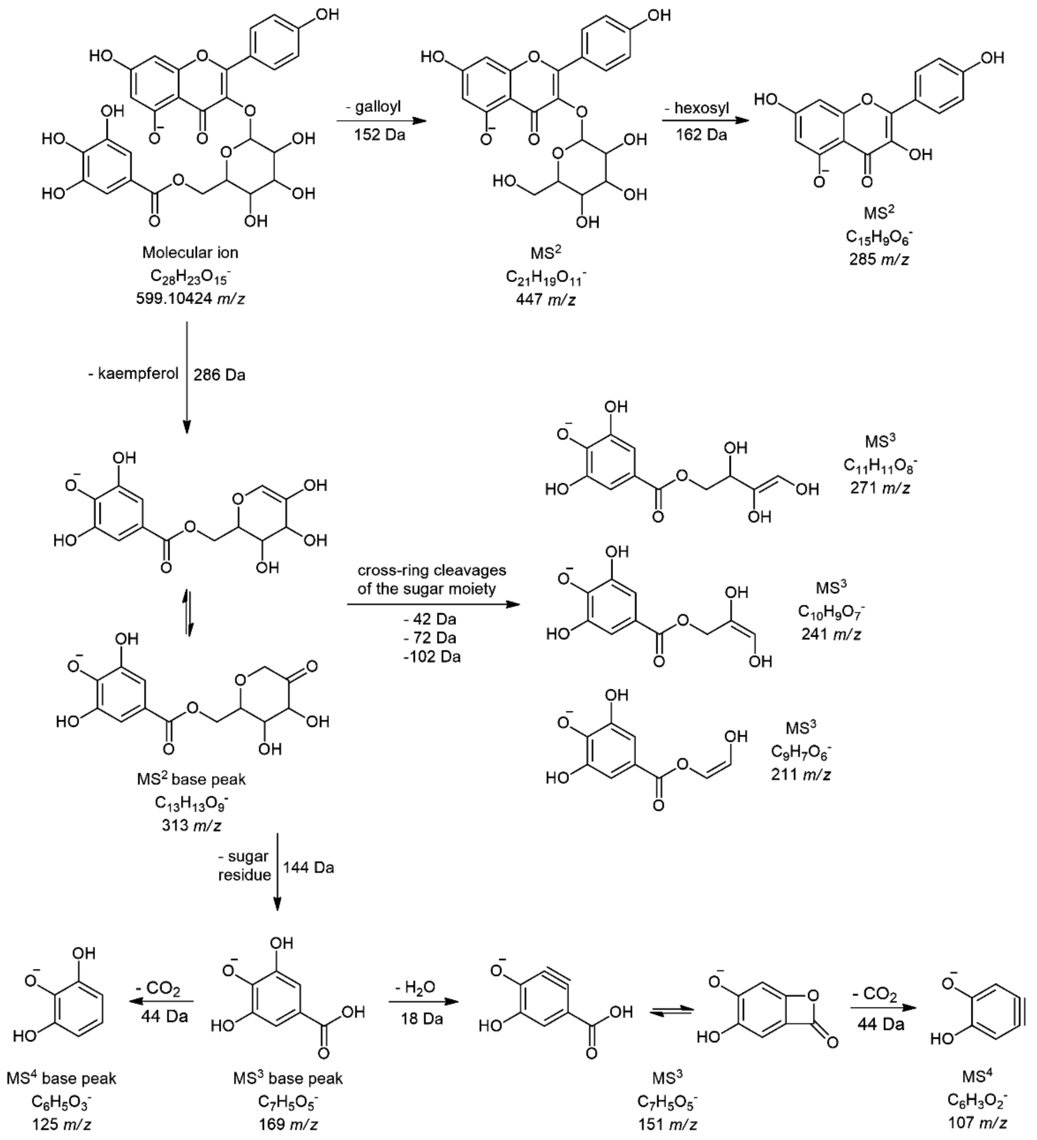

- galloyl
152 Da
- hexosyl
162 Da
Molecular ion
C28H23O15-
599.10424 m/z
MS2
C21H19O11-
447 m/z
MS2
C15H9O6-
285 m/z
- kaempferol
286 Da
MS3
C11H11O8-
271 m/z
cross-ring cleavages
of the sugar moiety
- 42 Da
- 72 Da
-102 Da
MS3
C10H9O7-
241 m/z
MS3
C9H7O6-
211 m/z
MS2 base peak
C13H13O9-
313 m/z
- sugar
residue
144 Da
- CO2
44 Da
- H2O
18 Da
- CO2
44 Da
MS4 base peak
C6H5O3-
125 m/z
MS3 base peak
C7H5O5-
169 m/z
MS3
C7H5O5-
151 m/z
MS4
C6H3O2-
107 m/z

Figure S1

Table S1

No	Compounds	Regression equation (Y = A + BX)		R^2	LOD, mg/L	LOQ, mg/L
		(A ± SE) × 10^5	(B ± SE) × 10^5			
**1**	**Gallic acid**	- 3.22 ± 1.11	72.56 ± 2.64	0.9961	0.06	0.21
**2**	**Gallocatechin**	- 5.41 ± 0.81	32.05 ± 1.57	0.9952	0.06	0.21
**3**	**Protocatechuic acid**	2.66 ± 1.36	223.63 ± 2.63	0.9993	0.03	0.10
**4**	**Aesculin**	- 0.16 ± 1.27	156.22 ± 2.66	0.9985	0.04	0.15
**5**	**Chlorogenic acid**	- 4.01 ± 1.69	94.49 ± 2.88	0.9963	0.08	0.27
**6**	**Catechin**	- 1.17 ± 1.43	112.93 ± 3.71	0.9957	0.06	0.21
**7**	***p*-Hydroxybenzoic acid**	8.35 ± 4.69	359.63 ± 10.99	0.9963	0.07	0.22
**8**	**Caffeic acid**	- 0.12 ± 1.20	223.33 ± 2.45	0.9994	0.03	0.10
**9**	**Syringic acid**	2.42 ± 3.34	236.76 ± 6.12	0.9967	0.08	0.25
**10**	**Vanillic acid**	2.84 ± 2.62	223.66 ± 5.28	0.9972	0.06	0.21
**11**	**Rutin**	- 3.43 ± 1.19	155.02 ± 2.10	0.9991	0.04	0.14
**12**	***p*-Hydroxyphenylacetic acid**	5.66 ± 2.58	180.38 ± 4.77	0.9965	0.08	0.26
**13**	**Hyperoside**	- 3.96 ± 1.43	129.05 ± 2.76	0.9977	0.06	0.20
**14**	***p*-Coumaric acid**	8.11 ± 4.37	313.16 ± 8.62	0.9962	0.08	0.25
**15**	**Catechin gallate**	- 9.18 ± 2.99	50.19 ± 3.11	0.9924	0.20	0.68
**16**	**Ferulic acid**	6.04 ± 5.97	251.15 ± 5.74	0.9974	0.13	0.43
**17**	**Myricetin**	0.11 ± 0.28	28.11 ± 0.73	0.9980	0.04	0.14
**18**	**Quercetin**	- 8.99 ± 7.31	183.45 ± 8.87	0.9927	0.18	0.60
**19**	**Naringenin**	- 8.30 ± 5.74	409.51 ± 9.70	0.9972	0.08	0.25
**20**	**Kaempferol**	- 6.94 ± 2.31	119.60 ± 7.62	0.9920	0.07	0.24

SE – standard error; LOD–limit of detection; LOQ–limit of quantification.
